# Pediatric heart failure classification based on left ventricular ejection fraction

**DOI:** 10.1002/pdi3.50

**Published:** 2023-12-27

**Authors:** Shan Huang, Xue Xiang, Xu Zhu, Jie Tian, Bo Pan, Min Zheng

**Affiliations:** ^1^ Department of Cardiology Children's Hospital of Chongqing Medical University Chongqing China; ^2^ Ministry of Education Key Laboratory of Child Development and Disorders Chongqing Key Laboratory of Child Infection and Immunity National Clinical Research Center for Child Health and Disorders China International Science and Technology Cooperation Base of Child Development and Critical Disorder Children's Hospital of Chongqing Medical University Chongqing China

**Keywords:** clinical features, left ventricle ejection fraction, pediatric heart failure, risk factors

## Abstract

Left ventricle ejection fraction (LVEF) is still not well acknowledged in classification of pediatric heart failure (PHF). We categorized PHF according to LVEF and aimed to determine the role of LVEF in PHF classification. Patients who were diagnosed with HF were divided into three groups according to their LVEF values: HF with reduced ejection fraction (HFrEF), HF with mildly reduced ejection fraction (HFmrEF), and HF with preserved ejection fraction (HFpEF). The clinical information of PHF patients was compared among those three groups. Factors associated with HF with improved EF (HFimpEF) and risk factors for in‐hospital death in PHF patients were analyzed. A total number of 1228 cases were collected. The proportion of HF patients with preserved LVEF (66.3%) was significantly higher than those with mildly reduced LVEF (21.7%) and reduced LVEF (12%). Clinical features such as age, B‐type natriuretic peptide (BNP) level, Ross classification, and E/A abnormal proportion in HF children with different LVEF value were statistically different. HF patients with younger age, lower BNP levels, minor cardiac dysfunction and less E/A abnormality could be found with higher LVEF value. The proportion of primary disease in PHF was largely different in HFpEF, HFmrEF and HFrEF groups. Medication treatment was more aggressive in patients with lower LVEF, except for vasoactive drugs. Children with congenital heart disease in HFrEF group were more prone to develop into HFimpEF. Sepsis, renal insufficiency, and an abnormal E/A ratio are risk factors for in‐hospital death of HF children. Clinical features of PHF could be well classified by LVEF, which is an essential and helpful indicator for PHF classification and management.

## Graphical abstract

Left ventricle ejection fraction is an essential and helpful indicator for PHF classification and management.

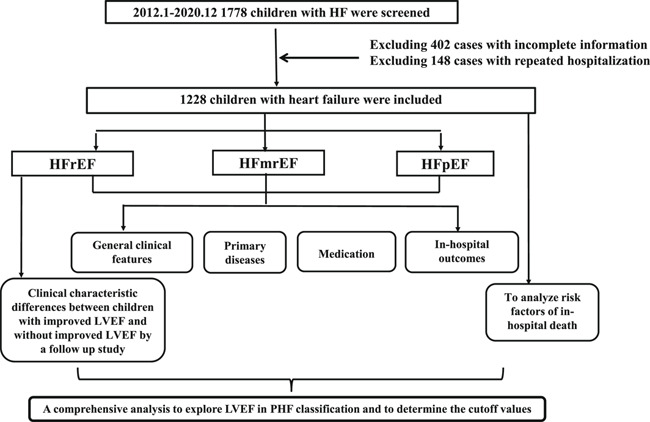

## 1 INTRODUCTION

Heart failure (HF) is a major threat to the global public health, affecting approximately 43 million people worldwide. In recent years, significant progress has been made in the epidemiology, diagnosis, and treatment of HF in adults.^1^ Owing to the frequent need for surgery or interventional therapy, the cost of HF in children is much higher than that in adults, which places a huge economic burden on society.^2^ However, the global epidemiology, causes, treatment strategies, and outcomes of HF in children remain largely unknown,^3^ and only data from local countries or regions have been reported. Studies have shown that the incidence of primary HF in children ranges from 0.87 per 100,000 (UK and Ireland) to 7.4 (Taiwan), and the prevalence of HF is approximately 83.3 per 100,000 (a large population‐based study in Spain).^4^

Left ventricular ejection fraction (LVEF) is an important indicator of HF and may play an important role in determining its pathophysiology and sensitivity to HF treatment.^1^ In recent years, patients with HF have been classified according to LVEF in many clinical guidelines, which have been updated several times.^5^, ^6^, ^7^ In 2021,^8^ the “Universal Definition and Classification of HF” issued by the Heart Failure Society of America (HFSA), Heart Failure Association of the European Society of Cardiology (HFA), and the Japanese Heart Failure Society suggested that patients with HF should be divided into four categories according to LVEF: HF with reduced ejection fraction (HFrEF): EF ≤40%; HF with mildly reduced ejection fraction (HFmrEF): LVEF ≥40% and LVEF ≤49%; HF with preserved ejection fraction (HFpEF): LVEF ≥50%. Studies have shown that^9^, ^10^ the clinical feature, treatment options and prognosis of adult patients with HF vary with LVEF. However, most of this evidence comes from adults, and the role of LVEF in pediatric HF patients remains controversial. At present, it is unclear whether pediatric heart failure (PHF) can be classified well by LVEF.

A LVEF category change in serial LVEF measurements is reportedly an important prognostic factor in Tohoku District‐2 (CHART‐2) Study and SwedeHF registries.^11^, ^12^ An increase in LVEF correlates with improved long‐term prognosis in patients with dilated cardiomyopathy.^13^ Conversely, LVEF reduction is an important indicator of poor outcomes in those with drug‐induced cardiomyopathy.^14^ Therefore, it is important to manage patients with HF by focusing on LVEF changes. In this study, the clinical features, pharmacological therapies, and risk factors for in‐hospital death in children with HF were analyzed. Children with HF were divided into three groups according to their LVEF value: HFrEF, HFmrE, and HFpEF. Our data demonstrates that LVEF is an useful indicator of PHF classification. Different types of PHF, which are classified based on LVEF, show various clinical features and medication treatment plans. We also followed up children with HFrEF for 6 months and summarized the characteristics of patients with an improved LVEF (≥10%).

## 2 METHODS

### 2.1 Study population

Hospitalized children diagnosed with HF for the first time at the Children's Hospital of Chongqing Medical University between January 2012 and December 2020 were studied. The diagnostic criteria for HF were based on “Recommendations for diagnosis and treatment of HF in children (2020 revised edition)”, the latest version. In brief, clinical manifestations (tachypnea, cyanosis, feeding difficulty, being fidgety, pale, fatigue, edema, liver enlargement, etc.), B‐type natriuretic peptide (BNP) levels, echocardiography, cMRI and heart function evaluation based on Ross classification were used to diagnose PHF.^15^, ^16^ Patients with incomplete main clinical data (such as lack of LVEF) were excluded. This study was approved by the ethics committee of the Children's Hospital of Chongqing Medical University (96/2021).

### 2.2 Data collection

Data for each patient were collected according to the electronic medical record system, including social demographic data, medical history, physical examination, Ross classification, primary causes of HF, LVEF, left ventricular stroke volume index (LVSVI), mitral E/A ratio, BNP, length of hospital stay, rate of treatment abandonment, and in‐hospital mortality. Ross classification standard was based on the Canadian Cardiovascular Society Guidelines.^15^ LVEF was measured by M mode using the Teichholz method. Mitral E/A <1 or >2 was defined as abnormal.

The methods of classifying children with HF based on LVEF values were as follows: according to the 2013 Canadian Cardiovascular Society Guidelines,^15^ children with EF <55% are considered to have abnormal systolic function, while adults with EF <50% are considered to have abnormal systolic function,^8^ suggesting that adults and children have different LVEF cutoff values. In this study, LVEF <55% was used as the cutoff value for abnormal left ventricular systolic function in children. Children with HF were divided into three groups according to LVEF value: HFrEF, EF ≤40%; HFmrEF, LVEF ≥41% and LVEF ≤54%; HFpEF, EF ≥55%. HFrEF patients were followed for 6 months, and LVEF improved more than 10% were collected in the HF with improved EF (HFimpEF) group.

### 2.3 Statistical analysis

Categorical variables were expressed as numbers (percentages), the chi‐square test or Fisher's exact probability analysis was used for comparison among groups, and the Bonferroni method was used to correct α for multiple comparisons. The mean value ± standard deviation was used for expression of normally distributed continuous variables, analysis of variance was used for comparison among groups. Continuous Skewed distribution variables were expressed as median (P25, P75). Wilcoxon test was used for comparison among groups, and Kruskal–Wallis one‐way ANOVA method was used for multiple comparisons. Risk factors were analyzed by binary logistic regression, and odds ratio (OR) was presented with 95% confidence intervals. IBMSPSS 25.0 software was used for data statistical analysis, and statistical significance was set at *p* < 0.05.

## 3 RESULT

### 3.1 Clinical features of HF children with different LVEF

From January 2012 to December 2020, 1778 children with HF were admitted to the Children's Hospital of Chongqing Medical University. After the exclusion criteria was implemented, 1228 cases were included in this study (Table 1). More than half of the children with HF were diagnosed with HFpEF (66.3%), 12.0% with HFrEF and 21.7% with HFmrEF, indicating that HFpEF might be the dominant type in pediatric HF patients. In our cohort, most of the children with HF were infants (68.7%), significantly more than preschool children (18.7%), school‐age children (8.2%), and adolescents (4.4%) (≥12 and <18 years old).

**TABLE 1:** Basic characteristics of children with HF with different LVEF.

	Total (*n* = 1228)	HFrEF (*n* = 148)	HFmrEF (*n* = 266)	HFpEF (*n* = 814)	*H*/*χ* ^2^ value	*p* value	Test
Proportion, *n* (%)		147 (12.0)	267 (21.7)	814 (66.3)			
Gender, *n* (%)							
Male	654 (53.3)	65 (43.9)	143 (53.8)	446 (54.8)^a^	5.981	0.05	Chi‐square test
Female	574 (46.7)	83 (56.1)	123 (46.2)	368 (45.2)			
Age (median [P25, P75]) (Y)	0.45 [0.15, 1.75]	0.98 [0.39, 5.40]	0.43 [0.13, 3.20]^a^	0.39 [0.13, 1.08]^a^	42.347	<0.001	Wilcoxon test
Classification of age, *n* (%)							
≤1 Y	843 (68.7)	75 (50.7)	167 (62.8)^a^	601 (73.8)^a^ ^,^ ^b^	41.056	<0.001	Chi‐square test
1–6 Y	230 (18.7)	40 (27.0)	54 (20.3)	136 (16.7)^a^			
7–12 Y	101 (8.2)	20 (13.5)	29 (10.9)	52 (6.4)^a^ ^,^ ^b^			
≥13 Y	54 (4.4)	13 (8.8)	16 (6.0)	25 (3.1)^a^			
Ross classification, *n* (%)							
II	642 (52.3)	48 (32.4)	120 (45.1)^a^	474 (58.2)^a^ ^,^ ^b^	100.72	<0.001	Chi‐square test
III	445 (36.2)	50 (33.8)	113 (42.5)	282 (34.6)			
IV	141 (11.5)	50 (33.8)	33 (12.4)^a^	58 (7.1)^a^			
Length of hospital stay (median [P25, P75]) (D)	13 [7, 24]	12 [7, 18]	15 [8, 27]^a^	12 [7, 23]^a^	9.932	0.007	Wilcoxon test
Hospitalization expenses (median [P25, P75]) (D)	20,030 [10,025, 59,902]	16,012 [9870, 29,890]	26,248 [12,123, 83,487]^a^	19,770 [9473, 58,034]^b^	18.177	<0.001	Wilcoxon test
BNP (median [P25, P75]) pg/mL	286 [89, 935]	896 [404, 2197]	360 [127, 1163]^a^	198 [62, 631]^a^ ^,^ ^b^	83.219	<0.001	Wilcoxon test
LVSVI (median [P25, P75]) mL/m^2^	52 [34, 78]	63 [43, 85]	50 [29, 74]^a^	51 [34, 78]^a^	12.59	0.002	Wilcoxon test
Abnormal E/A, *n* (%)	425 (35.2)	100 (68.5)	118 (45.2)^a^	207 (25.9)^a^ ^,^ ^b^	112.897	<0.001	Chi‐square test

Abbreviations: BNP, B‐type natriuretic peptide; D, day; E/A, E: peak mitral flow velocity in early diastole; A: peak mitral flow velocity in late diastole; HF, heart failure; HFmrEF, HF with mildly reduced ejection fraction; HFpEF, HF with preserved ejection fraction; HFrEF, HF with reduced ejection fraction; LVEF, left ventricular ejection fraction; LVSVI, left ventricular stroke volume index; Y, years old.

^a^ Compared with HFrEF, corrected *p* < 0.05.

^b^ Compared with HFmrEF, corrected *p* < 0.05.

Most children with HF in the HFpEF group were infants (73.8%), and the proportion of infants in the HFrEF (50.7%) and HFmrEF (62.8%) groups was significantly lower. Therefore, as illustrated in Table 1, the median age (years) in the three groups showed a decreasing trend as LVEF increased: HFrEF 0.98 (0.39, 5.40), HFmrEF 0.43 (0.13, 3.20), and HFpEF 0.39 (0.13, 1.08). This pattern was also found in BNP level (pg/mL), HFrEF 896 (404, 2197), HFmrEF 360 (127, 1163), and HFpEF 198 (62, 631), and the proportion of E/A abnormality, HFrEF 68.5%, HFmrEF 45.2%, and HFpEF 25.9%. Ross score is one of the most widely used and convincing indicators of cardiac function in patients with PHF. Our data showed that the heart function of 58.2% of HFpEF patients was level II, which was significantly higher than that of HFrEF patients (32.4%). In contrast, the proportion of children with level IV cardiac function in the HFrEF group was higher than that in HFpEF group (33.8% vs. 7.1%). Meanwhile, sex distribution also showed a significant difference between HFpEF and HFrEF patients, with more males in the HFpEF (54.8% vs. 43.9%) group than that in HFrEF group. These results suggest that clinical features, such as age, heart function, BNP level, and anomalous E/A ratio, were well classified in different LVEF‐driven types of PHF.

### 3.2 Primary causes in PHF patients with different LVEF

The distribution of the primary causes in the 1228 children with HF is shown in Table 2. There were 685 patients (55.8%) with congenital heart disease (CHD), 147 (12.0%) with cardiomyopathy, 91 (7.4%) with pneumonia, 85 (6.9%) with arrhythmia, 34 (2.8%) with renal insufficiency, 32 (2.6%) with neonatal respiratory distress syndrome (NRDS), 28 cases (2.3%) with sepsis, 27 cases (2.2%) with primary pulmonary hypertension (PPH), and 25 cases (2.0%) with myocarditis. These results suggest that CHD is the most common cause of HF in children, followed by cardiomyopathy, pneumonia, arrhythmia, renal insufficiency, NRDS, sepsis, PPH, and myocarditis.

**TABLE 2:** Etiology in children with HF with different LVEF.

	Total (*n* = 1228)	HFrEF (*n* = 148)	HFmrEF (*n* = 266)	HFpEF (*n* = 814)	*H*/*χ* ^2^ value	*p* value	Test
CHD, *n* (%)	685 (55.8)	20 (13.5)	130 (48.9)^a^	535 (65.7)^a^ ^,^ ^b^	144.976	<0.001	Chi‐square test
Cardiomyopathy, *n* (%)	147 (12.0)	97 (65.5)	37 (13.9)^a^	13 (1.6)^a^ ^,^ ^b^	487.123	<0.001	Chi‐square test
Pneumonia, *n* (%)	91 (7.4)	3 (2.0)	16 (6.0)	72 (8.8)^a^	9.448	0.009	Chi‐square test
Arrhythmia, *n* (%)	85 (6.9)	4 (2.7)	25 (9.4)^a^	56 (6.9)	6.624	0.036	Chi‐square test
Renal insufficiency, *n* (%)	34 (2.8)	6 (4.1)	13 (4.9)	15 (1.8)^b^	7.935	0.019	Chi‐square test
ARDS, *n* (%)	32 (2.6)	1 (0.7)	5 (1.9)	26 (3.2)	3.835	0.147	Chi‐square test
Sepsis, *n* (%)	28 (2.3)	0 (0.0)	6 (2.3)	22 (2.7)	4.106	0.128	Chi‐square test
PPH, *n* (%)	27 (2.2)	0 (0.0)	1 (0.4)	26 (3.2)^b^	11.188	0.004	Chi‐square test
Myocarditis, *n* (%)	25 (2.0)	6 (4.1)	12 (4.5)	7 (0.9)^a^ ^,^ ^b^	16.839	<0.001	Chi‐square test
AIRD, *n* (%)	17 (1.4)	0 (0.0)	6 (2.3)	11 (1.4)	3.324	0.17	Fisher's exact
Coronary artery disease, *n* (%)	18 (1.5)	7 (4.7)	5 (1.9)	6 (0.7)^a^	11.627	0.002	Fisher's exact
Mechanical factors, *n* (%)	12 (1.0)	2 (1.4)	4 (1.5)	6 (0.7)	1.98	0.36	Fisher's exact
Valve dysfunction, *n* (%)	9 (0.7)	0 (0.0)	4 (1.5)	5 (0.6)	2.689	0.243	Fisher's exact
Others, *n* (%)	18 (1.5)	2 (1.4)	3 (1.1)	13 (1.6)	0.224	0.935	Fisher's exact

Abbreviations: AIRD, autoimmune rheumatic disease; ARDS, acute respiratory distress syndrome; CHD, congenital heart disease; HF, heart failure; HFmrEF, HF with mildly reduced ejection fraction; HFpEF, HF with preserved ejection fraction; HFrEF, HF with reduced ejection fraction; LVEF, left ventricular ejection fraction; PPH, primary pulmonary hypertension.

^a^ Compared with HFrEF, corrected *p* < 0.05.

^b^ Compared with HFmrEF, corrected *p* < 0.05.

Compared to HFrEF group, the proportion of CHD in the HFpEF group was higher (HFpEF vs. HFrEF, 65.7% vs. 13.5%), and cardiomyopathy (HFpEF vs. HFrEF, 1.6% vs. 65.5%), myocarditis (HFpEF vs. HFrEF, 0.9% vs. 4.1%), and coronary artery disease (HFpEF vs. HFrEF, 0.7% vs. 4.7%) were lower (*p* < 0.05). These data suggest that the etiology composition in the HFrEF group was largely different from that in the HFpEF group. It is not surprising that the proportion of CHD (48.9%) and cardiomyopathy (13.9%) in primary disease of HFmrEF was in between. However, it is worth noting that these differences (CHD ratio and cardiomyopathy ratio) between HFmrEF and HFpEF or between HFmrEF and HFrEF were all statistically significant. Taken together, the classification of PHF based on LVEF could be a rational way, as the dominant primary diseases of PHF are well organized. For other primary diseases, we failed to find an orderly pattern of HFmrEF between HFpEF and HFrEF patients. This might be caused by a small number of cases, and/or the cutoff value of LVEF in the definition of HFmrEF is not sufficiently solid, as there are currently few reports on this concept in PHF.

### 3.3 Pharmacologic therapy for HF in children with different LVEF

Medications used in the 1228 patients with PHF mainly included six categories (as shown in Table 3): angiotensin‐converting enzyme inhibitors (ACEI) (59.9%), diuretics (92.4%), vasoactive drugs (26.9%), positive inotropic drugs (84.9%), β‐blockers (10.3%), and myocardial energy metabolizers (87.3%). The usage rates of ACEI, diuretics, inotropic agents, beta‐blockers, and myocardial energy metabolizers in HFrEF patients were significantly higher than those in HFpEF children. When comparing the HFmrEF and HFpEF groups, the use of positive inotropic drugs, β‐blockers, and myocardial energy metabolizers was higher in the HFmrEF group. The usage rates of ACEI and diuretics in the HFmrEF group were lower than those in the HFrEF group. These data indicate that pediatricians tend to select more aggressive pharmacologic therapy plans in patients with lower LVEF values.

**TABLE 3:** Pharmacologic therapy for HF in children with different LVEF.

	Total (*n* = 1228)	HFrEF (*n* = 148)	HFmrEF (*n* = 266)	HFpEF (*n* = 814)	*χ* ^2^ value	*p* value	Test
Diuretic, *n* (%)	1135 (92.4)	145 (98.0)	244 (91.7)^a^	746 (91.6)^a^	7.397	0.025	Chi‐square test
Furosemide	1053 (85.7)	129 (87.2)	225 (84.6)	699 (85.9)	0.546	0.761	Chi‐square test
Hydroflumethiazide	564 (45.9)	81 (54.7)	128 (48.1)	355 (43.6)^a^	6.89	0.032	Chi‐square test
Spirolactone	883 (72.0)	126 (85.1)	194 (73.2)^a^	563 (69.2)^a^	16.09	<0.001	Chi‐square test
Myocardial energy metabolism drugs, *n* (%)	1072 (87.3)	142 (95.9)	253 (95.1)	677 (83.2)^a^ ^,^ ^b^	37.14	<0.001	Chi‐square test
Coenzyme	325 (26.5)	53 (35.8)	69 (25.9)	203 (24.9)^a^	7.655	0.022	Chi‐square test
Levocarnitine	269 (21.9)	59 (39.9)	71 (26.7)^a^	139 (17.1)^a^ ^,^ ^b^	42.564	<0.001	Chi‐square test
Creatine phosphate sodium	999 (81.4)	136 (91.9)	238 (89.5)	625 (76.8)^a^ ^,^ ^b^	33.613	<0.001	Chi‐square test
Fructose dipllosphate	112 (9.1)	29 (19.6)	33 (12.4)	50 (6.1)^a^ ^,^ ^b^	31.762	<0.001	Chi‐square test
Positive inotropic agents, *n* (%)	1042 (84.9)	141 (95.3)	244 (91.7)	657 (80.7)^a^ ^,^ ^b^	33.14	<0.001	Chi‐square test
Digoxin	701 (57.1)	113 (76.4)	160 (60.2)^a^	428 (52.6)^a^	30.189	<0.001	Chi‐square test
Dopamine	706 (57.5)	78 (52.7)	166 (62.4)	462 (56.8)	4.197	0.123	Chi‐square test
Dobutamine	476 (38.8)	71 (48.0)	111 (41.7)	294 (36.1)^a^	8.674	0.013	Chi‐square test
Epinephrine	500 (40.7)	43 (29.1)	138 (51.9)^a^	319 (39.2)^b^	22.859	<0.001	Chi‐square test
Norepinephrine	156 (12.7)	12 (8.1)	45 (16.9)^a^	99 (12.2)	7.292	0.026	Chi‐square test
Milrinone	682 (55.5)	94 (63.5)	169 (63.5)	419 (51.5)^a^ ^,^ ^b^	16.143	<0.001	Chi‐square test
ACEI, *n* (%)	736 (59.9)	120 (81.1)	162 (60.9)^a^	454 (55.8)^a^	33.533	<0.001	Chi‐square test
Vasoactive drugs, *n* (%)	330 (26.9)	23 (15.5)	78 (29.3)^a^	229 (28.1)^a^	11.142	0.004	Chi‐square test
Sodium nitroprusside	82 (6.7)	3 (2.0)	25 (9.4)^a^	54 (6.6)	8.299	0.016	Chi‐square test
Nitroglycerin	61 (5.0)	7 (4.7)	21 (7.9)	33 (4.1)^b^	6.285	0.043	Chi‐square test
Phentolamine	246 (20.0)	15 (10.1)	55 (20.7)^a^	176 (21.6)^a^	10.402	0.006	Chi‐square test
β‐blocker, *n* (%)	126 (10.3)	30 (20.3)	44 (16.5)	52 (6.4)^a^ ^,^ ^b^	40.757	<0.001	Chi‐square test

Abbreviations: ACEI, angiotensin‐converting enzyme inhibitors; HF, heart failure; HFmrEF, HF with mildly reduced ejection fraction; HFpEF, HF with preserved ejection fraction; HFrEF, HF with reduced ejection fraction; LVEF, left ventricular ejection fraction.

^a^ Compared with HFrEF, corrected *p* < 0.05.

^b^ Compared with HFmrEF, corrected *p* < 0.05.

### 3.4 In‐hospital outcomes of children with HF with different LVEF

Of the 1228 children with HF, 121 (9.9%) died during hospitalization (Table 4). There was no statistically significant difference in in‐hospital mortality among the HFrEF (8.9%), HFmrEF (10.5%), and HFpEF (9.8%) groups. The treatment abandonment rate of each group was not statistically different. In our cases, treatment abandonment included two conditions: (1) patients refused further rescue (DNR), and (2) patients discharged without a sufficient course of treatment.

**TABLE 4:** In‐hospital outcomes of children with HF with different LVEF.

	Total (*n* = 1228)	HFrEF (*n* = 148)	HFmrEF (*n* = 266)	HFpEF (*n* = 814)	*χ* ^2^ value	*p* value	Test
In‐hospital death	121 (9.9)	13 (8.8)	28 (10.5)	80 (9.8)	0.327	0.849	Chi‐square test
Treatment abandonment	299 (24.3)	41 (27.7)	71 (26.7)	187 (23.0)	2.533	0.282	Chi‐square test
DNR	46 (3.7)	2 (1.4)	10 (3.8)	34 (4.2)	2.773	0.25	Chi‐square test
Discharged without sufficient course of treatment	253 (20.6)	39 (26.4)	61 (22.9)	153 (18.8)	5.497	0.064	Chi‐square test

Abbreviations: DNR, do not resuscitate; HF, heart failure; HFmrEF, HF with mildly reduced ejection fraction; HFpEF, HF with preserved ejection fraction; HFrEF, HF with reduced ejection fraction; LVEF, left ventricular ejection fraction.

### 3.5 Analysis of risk factors for in‐hospital death in PHF patients

We then compared the differences in the primary diseases, proportion of abnormal E/A ratio and levels of BNP and LVSVI between patients who died or survived in‐hospital. Statistically different factors, age, sex, and the three groups divided according to LVEF value, were entered into a binary logistic regression analysis as independent variables, and in‐hospital death was defined as the dependent variable.

Data in Table 5 show that sepsis (OR = 6.559), renal insufficiency (OR = 4.351) and an abnormal E/A ratio (OR = 3.275) increased the risk of in‐hospital death in children with HF. LVEF was not an independent risk factor for in‐hospital mortality, after adjusting for confounding factors.

**TABLE 5:** Analysis of risk factors for in‐hospital death in children with HF.

Variable	*B*	SE	Wald	*p* value	OR (95% CI)
Renal insufficiency	1.47	0.39	14.207	<0.001	4.351 (2.026–9.347)
Abnormal E/A	1.186	0.302	15.442	<0.001	3.275 (1.812–5.918)
LVSVI	−0.029	0.006	22.458	<0.001	0.971 (0.96–0.983)
Sepsis	1.881	0.608	9.555	0.002	6.559 (1.99–21.618)
Cardiomyopathy	−0.793	0.546	2.103	0.147	0.453 (0.155–1.321)
Arrhythmia	−0.647	0.462	1.961	0.161	0.524 (0.212–1.295)
ARDS	−0.948	1.101	0.741	0.389	0.388 (0.045–3.354)
Malnutrition	−0.366	0.52	0.497	0.481	0.693 (0.25–1.92)
BNP	0	0	0.437	0.509	1.000 (1.000–1.000)
Valve dysfunction	−0.135	0.33	0.167	0.682	0.874 (0.457–1.669)
HFrEF (control)			1.64	0.44	
HFmrEF	−0.613	0.517	1.407	0.235	0.541 (0.197–1.492)
HFpEF	−0.267	0.487	0.301	0.584	0.766 (0.295–1.99)
Age	−0.063	0.038	2.767	0.096	0.939 (0.871–1.011)
Gender	0.067	0.285	0.055	0.814	1.069 (0.611–1.871)

Abbreviations: ARDS, acute respiratory distress syndrome; BNP, B‐type natriuretic peptide; CI, confidence intervals; E/A, E: peak mitral flow velocity in early diastole; A: peak mitral flow velocity in late diastole; HF, heart failure; HFmrEF, HF with mildly reduced ejection fraction; HFpEF, HF with preserved ejection fraction; HFrEF, HF with reduced ejection fraction; LVSVI, left ventricular stroke volume index; OR, odds ratios; SE, standard error.

### 3.6 Characteristics of HFrEF patients with and without improved EF

After 6 months of follow‐up, HFimpEF patients were compared with those who's EF fail to increase by ≥10%. As shown in Table 6, HFimpEF patients were younger (0.67 [0.39, 2.79] vs. 4.03 [0.87, 11.35] years), with minor Ross classification (level II, 39.7% vs. 22.2%, and level IV, 20.6% vs. 44.4%), compared with patients without improved EF.

**TABLE 6:** Characteristics of HFrEF patients with and without improved EF.

	Total (*n* = 117)	Without improved (*n* = 54)	With improved (*n* = 63)	*Z*/*χ* ^2^ value	*p* value	Test
Age (median [P25, P75]) (Y)	1.25 [0.51, 5.92]	4.03 [0.87, 11.35]	0.67 [0.39, 2.79]	−3.414	0.001	Mann–Whitney
Gender, *n* (%)						
Male	46 (39.3)	21 (38.9)	25 (39.7)	0.008	0.93	Chi‐square test
Female	71 (60.7)	33 (61.1)	38 (60.3)			
Ross classification, *n* (%)						
II	37 (31.6)	12 (22.2)	25 (39.7)*	8.334	0.015	Chi‐square test
III	43 (36.8)	18 (33.3)	25 (39.7)			
IV	37 (31.6)	24 (44.4)	13 (20.6)*			
Length of hospital stay (median [P25, P75]) (D)	13 [9, 21]	13 [8, 18]	14 [10, 23]	−1.429	0.153	Mann–Whitney
BNP (median [P25, P75]) pg/mL	804 [382, 1959]	908 [539, 2378]	718 [253, 1669]	−1.694	0.09	Mann–Whitney
LVSVI (median [P25, P75]) mL/m^2^	71 [53, 91]	72 [55, 92]	69 [46, 90]	−0.596	0.551	Mann–Whitney
Abnormal E/A, *n* (%)	79 (67.5)	41 (75.9)	38 (60.3)	3.230	0.072	Chi‐square test
Primary disease, *n* (%)						
Cardiomyopathy	85 (72.6)	43 (79.6)	42 (66.7)	2.459	0.117	Chi‐square test
CHD	9 (7.7)	3 (5.6)	6 (9.5)	0.207	0.649	Chi‐square test
Coronary artery disease	7 (6.0)	2 (3.7)	5 (7.9)	0.327	0.568	Chi‐square test
Myocarditis	4 (3.4)	2 (3.7)	2 (3.2)	0.000	1	Chi‐square test
Arrhythmia	4 (3.4)	1 (1.9)	3 (4.8)	0.125	0.724	Chi‐square test
Renal insufficiency	4 (3.4)	2 (3.7)	2 (3.2)	0.000	1	Chi‐square test
Pneumonia	1 (0.9)	0 (0.0)	1 (1.6)		1	Fisher's exact
Others	1 (0.9)	1 (1.9)	0 (0.0)		0.938	Fisher's exact

Abbreviations: BNP, B‐type natriuretic peptide; CHD, congenital heart disease; E/A, E: peak mitral flow velocity in early diastole; A: peak mitral flow velocity in late diastole; EF, ejection fraction; HFrEF, HF with reduced ejection fraction; LVSVI, left ventricular stroke volume index.

*p < 0.05.

## 4 DISCUSSION

In this study, the clinical features and risk factors for in‐hospital death of children with HF in our hospital were analyzed, and patients were grouped according to LVEF value for the first time, referring to adult HF classification. Our data indicated that HFpEF (66.3%) is the dominant type of PHF, accompanied by younger age, milder heart function, and lower BNP levels. In contrast, 12.0% of patients with PHF were classified in the HFrEF group, with older age, more severe cardiac dysfunction, and a much higher BNP level. The children in the HFmrEF group had an in‐between condition. Interestingly, our data indicated a higher E/A abnormal proportion in HFrEF patients compared with HFpEF children, which is different from adult conditions, as diastolic dysfunction is necessary to diagnose HFpEF in adults. This does not mean that the cardiac diastolic function of children with HFpEF might be superior to that of adults. We assume that in children with HF, the E/A ratio might not be a suitable or an early marker for pediatric patients. Our ongoing prospective research indicates low sensitivity of E/A ratio in the diagnosis of heart diastolic dysfunction (data not published). In addition, in children with HFrEF, both systolic and diastolic heart functions are more likely to be diminished to a great extent.

The common etiology of HF in adults are coronary heart disease, hypertension, valvular disease, arrhythmia and cardiomyopathy.^9^, ^17^ Studies have shown that the underlying disease spectrum of HF in children is different from that in adults, with CHD and cardiomyopathy being the most common primary disease.^18^, ^19^, ^20^ Shaddy et al.^4^ conducted a systematic review of literature on HF in children from 1996 to 2016. They found that the main causes of HF in children are cardiomyopathy, CHD, parasitic infections, lower respiratory tract infections, malnutrition, severe anemia, and rheumatic heart disease. A study of 67,349 children with HF in the United States showed that 87% had CHD and 6% had cardiomyopathy.^20^ In the present study, we found that the main causes of HF in children were CHD, cardiomyopathy, pneumonia, and arrhythmia, which is consistent with previous studies. Data from the present study indicate that the proportion of primary diseases of different types of PHF were largely different. Taken together, the clinical features, types of primary disease, and pharmacological treatment plan of PHF could be well classified according to LVEF.

Studies on adult HF patients found that HFmrEF was heterogeneous and derived from HFrEF patients with improved EF or HFpEF with decreased EF.^21^, ^22^, ^23^ Our study found that the clinical features of children with HFmrEF, including age, severity of HF symptoms, BNP level, and etiology composition showed an in‐between condition, indicating that children with HFmrEF may also be derived from patients with HFrEF and HFpEF.

Studies in the United States have indicated that the in‐hospital all‐cause mortality of children with HF ranged from 6.3% to 7.4%, and many comorbid conditions, such as arrhythmia, pulmonary hypertension, sepsis, and renal failure are risk factors for in‐hospital death.^3^, ^24^ Our study suggests that the all‐cause mortality of children with HF was 9.9%. Sepsis, renal insufficiency, and abnormal E/A ratio are risk factors for in‐hospital mortality. The mortality rate among the three different groups of HF patients is quite similar. Here, the mortality rate was referred to in hospital death rate, which cannot stand for the long‐term mortality of HF. Meanwhile, patients discharged without a sufficient course of treatment also induced a bias in analysis of in‐hospital mortality. Most of those children with HF were chronic, discharge follow‐up with longer terms would be of great value in study of survival rate of PHF children.

Multiple observational studies have shown that the risk of death in adults with HFpEF is as high as that in adults with HFrEF^10^, ^25^, ^26^. However, an adjusted meta‐analysis showed that HFpEF patients had a 32% lower risk of death than HFrEF patients.^1^, ^27^ Our study found that there was no statistically difference in in‐hospital mortality between children with HFrEF and HFpEF. This is not surprising, because we only showed the in‐hospital mortality of PHF patients, and a long‐term follow‐up is strongly needed to compare mortality in different types of PHF. The treatment abandonment rate in patients with HFrEF was higher than that in patients with HFpEF, indicating that children with HFrEF might require a longer treatment course in PHF.

## 5 CONCLUSION

The present study suggests that PHF can be well classified based on LVEF, and this classification is helpful in management of patients with PHF. This was a single‐center retrospective study with a small sample size, in which bias may exist. Also, in the primary cause part, as the limitation of retrospective study, those basic diseases which were causes or just complications of HF could not be well distinguished. Therefore, a multicenter, large‐sample, prospective study is planned to further improve the reliability of this study.

## AUTHOR CONTRIBUTIONS

Dr Min Zheng, Bo Pan, and Jie Tian contributed to the conception and design of the study. Dr Min Zheng, Shan Huang, Xue Xiang, and Xu Zhu organized the data and performed the statistical analyze. Dr Bo Pan, Shan Huang, and Min Zheng drafted the manuscript. Dr Bo Pan and Min Zheng wrote the sections of the manuscript. All authors approved the final manuscript as submitted and agree to be accountable for all aspects of the work.

## CONFLICT OF INTEREST STATEMENT

Jie Tian is the Deputy Editor‐in‐Chief of Pediatric Discovery. To minimize bias, he was excluded from all editorial decision‐making related to the acceptance of this article for publication. The authors have no conflicts of interest relevant to this article to disclose.

## ETHICS STATEMENT

This study was approved by the ethics committee of the Children's Hospital of Chongqing Medical University (96/2021).

## Data Availability

All datasets presented in this study are included in the article files.
